# Identifying rheumatoid arthritis susceptibility genes using high-dimensional methods

**DOI:** 10.1186/1753-6561-3-s7-s79

**Published:** 2009-12-15

**Authors:** Xueying Liang, Ying Gao, Tram K Lam, Qizhai Li, Cathy Falk, Xiaohong R Yang, Alisa M Goldstein, Lynn R Goldin

**Affiliations:** 1 Division of Cancer Epidemiology and Genetics, National Cancer Institute, National Institutes of Health, 6120 Executive Boulevard, Bethesda, Maryland 20892, USA; 2 Cancer Prevention Fellowship Program, Office of Preventive Oncology, National Cancer Institute, National Institutes of Health, Bethesda, Maryland 20852, USA; 3 Academy of Mathematics and Systems Science, Chinese Academy of Sciences, Beijing 100190, PR China; 4 Teaneck, New Jersey, USA

## Abstract

Although several genes (including a strong effect in the human leukocyte antigen (HLA) region) and some environmental factors have been implicated to cause susceptibility to rheumatoid arthritis (RA), the etiology of the disease is not completely understood. The ability to screen the entire genome for association to complex diseases has great potential for identifying gene effects. However, the efficiency of gene detection in this situation may be improved by methods specifically designed for high-dimensional data. The aim of this study was to compare how three different statistical approaches, multifactor dimensionality reduction (MDR), random forests (RF), and an omnibus approach, worked in identifying gene effects (including gene-gene interaction) associated with RA. We developed a test set of genes based on previous linkage and association findings and tested all three methods. In the presence of the HLA shared-epitope factor, other genes showed weaker effects. All three methods detected SNPs in *PTPN22 *and *TRAF1-C5 *as being important. But we did not detect any new genes in this study. We conclude that the three high-dimensional methods are useful as an initial screening for gene associations to identify promising genes for further modeling and additional replication studies.

## Introduction

Rheumatoid arthritis (RA) is a chronic inflammatory disease affecting approximately 1% of the population [1]. Studies suggest that both genetic and environmental factors contribute to susceptibility to RA, with an estimated heritability of 60% [2]. Although *HLA-DRB1 *and *PTPN22 *are two genes that have been consistently associated with RA, they do not account for all of the genetic variation in RA [3]. Recent genome-wide association studies identified *TRAF1-C5 *and a locus on chromosome 1q13 as being associated with RA [4,5]. It is unknown whether gene-gene interactions play a role in disease etiology. Several methods have been designed specifically to conduct analyses of high-dimensional problems, including multifactor dimensionality reduction (MDR) [6], random forests (RF) [7,8], and an omnibus approach [9]. These approaches are currently exploratory but they permit testing for gene-gene interaction effects. In this study, we applied these three approaches as well as logistic regression to Genetic Analysis Workshop 16 (GAW16) data specifically to test their ability to detect genes associated with RA. We chose a limited set of single-nucleotide polymorphisms (SNPs) based on previous linkage and association results to make the data set more defined and more likely to be enriched for genes with true effects.

## Methods

### Data set

We used the GAW16 RA data set, which contains 545,080 SNPs genotyped on 868 RA cases and 1194 controls. The detailed sample and genotyping information are described elsewhere [4]. All the phenotypes, covariates, and genotypes for regions of interest were extracted from the overall data set. We limited our analyses to selected genes and regions identified from previous studies. The SNP list was selected based on genes/regions identified from a whole-genome linkage scan using the North American Rheumatoid Arthritis Consortium (NARAC) families [10], genome-wide association studies using these same case-control data [4], and the Wellcome Trust Case Control Consortium (WTCCC) data, which includes an RA subset [5] (Table 1). We selected 100 SNPs flanking each linkage peak marker (rs1354905 in chromosome 2 with LOD = 3.52 and rs2035693 in chromosome 11 with LOD = 3.09) [10]. Six genes/regions were identified from association studies by selecting the top three findings in Plenge et al. [4] and Wellcome Trust RA study [5]. Each gene was flanked by 50 kb up- and down-stream to incorporate potential regulatory elements. All available markers in these regions were identified and extracted from the GAW16 data set.

**Table 1:** Selected genes and regions

Source	Gene/region	Chromosome	Location (Mb)	No. selected SNPs	**Ref**.
Association study	*PTPN22*	1	114.0-114.2	21	[4]
	*TRAF1-C5*	9	120.7-121.0	38	[4]
	*CD40*	20	44.1-44.2	30	[4]
	chr 1	1	114.0-114.1	11	[5]
	chr 7	7	130.8-130.9	20	[5]
	chr 10	10	6.0-6.2	65	[5]
Linkage study	chr 2	2	191.8-192.3	100	[10]
	chr 11	11	40.7-41.2	101	[10]
					
Total				386 (378)^a^	

^a^8 SNPs on chromosome 1 overlapped between two studies; therefore 378 SNPs were in the final list.

### Data analysis

The initial quality control screening of all SNPs was performed using the Hardy-Weinberg equilibrium test in the computer program Haploview, separately in cases and controls. We also examined linkage disequilibrium patterns in each of the selected genes and regions because markers that are in high linkage disequilibrium will cause computational problems for some methods [11]. We ran Tagger [12] using different linkage disequilibrium levels to evaluate the tagSNPs among our selected markers and then set a criterion of *r*^2 ^< 0.5 to select tagSNPs for the final analysis. A single-SNP allelic chi-squared test for association was also computed using Haploview. The HLA shared-epitope (SE) status was included in the analyses.

We applied three high-dimensional methods to the selected SNPs: 1) MDR, 2) omnibus, and 3) RF. MDR is a data reduction approach for detecting and characterizing multi-locus genotype combinations that interact to predict disease risk for common, complex disease. It pools genotypes into "high-risk" and "low-risk" groups in order to reduce multidimensional data into only one dimension [6]. For the MDR analysis, we conducted analyses with and without the SE in the model. Five-fold cross-validation and 1000 permutations were performed to determine the statistical significance level.

The omnibus method tests for gene-based effects by considering all SNPs in the gene/region as a single group and evaluates the "test" gene assuming a known gene or other risk factor plays a role [9]. This method uses a logistic regression approach but the significance of the test gene effect includes both the main effect and the interaction between this gene and the known risk factor or gene. For this analysis, SE was included as the known risk gene for each of the test loci. For the genes identified by these methods, logistic regression was used to formally test whether the interaction terms were significant predictors.

The RF method is a tree-based classification and regression method. It uses two measures for SNP importance: mean decrease accuracy (MDA), which permutes the values of each variable in the out-of-bag cases and records the prediction, and mean decrease Gini (MDG), which measures the quality of a split in every node of the trees. We used the RF package for the statistical package R, and based the analysis on the classification method [7,8]. For each run we grew 500 trees. Runs with 1000 and 5000 trees were also carried out with very similar results (results not shown). We started with all SNPs and SE status and identified those factors with the greatest influence on disease phenotype, based on the RF measure of importance. We arbitrarily selected the top 15 SNPs and SE status for further study. The classification error rates were used as a measure of how well the RF predicted disease status. We started by looking at all pairs of factors and compared the percentage of records correctly classified by the generated RF for each pair. Any pair that stood out with a markedly lower classification error rate would indicate interaction. We also generated RFs based on a growing set of factors, adding them one at a time from the ordered importance list and then again compared the classification error rates.

## Results

Of the 378 markers selected, 20 were excluded for the following reasons: not polymorphic (*n *= 6), minor allele frequency ≤ 0.1% (*n *= 8), and not in Hardy-Weinberg equilibrium in controls (*n *= 6, *p *< 0.0001). Results from single-SNP allelic chi-square association test showed 86 SNPs significantly associated with RA (*p *< 0.05). Of these, one SNP on *PTPN22*, rs2476601, was highly associated with RA (*p *< 10^-12^). Consistent with findings from the study of Plenge et al. [4], statistical significance was observed for 10 SNPs in *TRAF1-C5 *(*p*-values ranged from 10^-5 ^to 10^-9^) as well as one SNP in *CD40 *(rs1569723, *p *< 10^-5^). Interestingly, we observed that SNP rs1517853 in the linkage region on chromosome 2, was also significant at *p *< 10^-4^. This marker is 37.8 kb away from the SNP at the linkage peak (rs1949429, LOD = 3.52). However, the peak SNP was not in the GAW16 data set. After adjusting for SE, statistical significance was observed for only rs2476601 in *PTPN22 *and rs3789597 in *RSBN1*/chromosome 1 (OR = 1.62 with 95% CI = 1.09~2.39) from logistic regression. A total of 175 tagSNPs were selected at an *r*^2 ^< 0.5 level to represent the 378 selected SNPs across the eight genes/regions.

Table 2 shows the best models for these genes in RA using the MDR approach. SE status was the strongest risk factor identified, with an average prediction accuracy of 76.24% (*p *< 0.0001). When we allowed for two genes in the analysis, a marker from the chromosome 2 linkage peak (rs1517835) was identified in addition to SE and predicted disease status correctly 75.54% of the time (*p *< 0.0001). However, the cross validation was low (40%) and the overall prediction rate was not higher than SE alone. Because SE is such a strong risk factor, it might mask weaker effects conferred by other markers. We therefore conducted additional MDR analyses excluding SE from the model. *PTPN22 *(rs2476601) was identified as the best single-locus model. In a two-locus model, MDR identified *PTPN22 *and *TRAF1-C5 *(p < 0.0001) with a predicted accuracy of 57.64%, but this is only a slight improvement over the one-locus model.

**Table 2:** Best models detected by MDR analyses

No. locus/loci in model	Best model	Accuracy (%)	Cross validation	*p*-Value
With SE				
1-locus	SE	76.24	100%	< 0.0001
2-locus	SE chr 2 linkage (rs1517835)	75.54	40%	< 0.0001
				
Without SE				
1-locus	*PTPN22 *(rs2476601)	56.27	100%	< 0.0001
2-locus	*PTPN22 *(rs2476601) *TRAF1-C5 *(rs3761847)	57.64	100%	< 0.0001

Table 3 shows the results from the omnibus method. With SE as known risk gene in the model, the omnibus method identified the *TRAF1-C5 *gene as having the strongest gene effect on RA (*p *= 0.0006, this *p*-value reflects both the main effect and the interaction between *TRAF1-C5 *and SE). Similarly, *PTPN22 *showed a gene effect with *p *= 0.0018. However, when we used logistic regression to validate this two-locus model, no interaction was observed for these two genes (*p *= 0.55), and they did not show a significant interaction with SE.

**Table 3:** *p*-Value for each gene/region considering SE as known risk gene in the omnibus method

Gene 1	No. SNPs	Score test	*p*-Value
chr 1/*PTPN22*	9	37.48	**0.0018**
chr 2/linkage	49	49.07	0.8957
chr 7	11	17.04	0.3417
chr 9/*TRAF1-C5*	13	44.01	**0.0006**
chr 10	33	61.15	0.1100
chr 11/linkage	41	44.96	0.7077
chr 20/*CD40*	19	32.22	0.1920

^a ^Bold font indicates *p *< 0.05.

As with the MDR analysis, the RF method showed that SE status was the most important risk factor in RA because it correctly classified disease status of individuals 72% of the time. Interestingly, SE status was more accurate at predicting which individuals had RA (error rate ~2%) than controls (error rate 47%). By incrementally adding SNPs according to importance, we found that the error rate reached a minimum with SE status and the top eight SNPs (Table 4). With these factors, 77% of individuals were correctly classified (73% of RA individuals and 80% of controls). The odd imbalance of false positives and negatives seen with SE status alone disappeared with the addition of the top eight SNPs. The top nine factors identified by MDA importance are shown in Table 4. For comparison, we also show the importance ranking by MDG. As with the MDR and omnibus approach, SNPs in *PTPN22 *and *TRAF1-C5 *were in the top set along with other SNPs, particularly in two candidate genes, *IL2RA *and *CD40*.

**Table 4:** Detection of important factors using RF

			Ranking by importance	
			
Marker	Gene	Chr	MDA	MDG
SE	Shared epitope	N/A	1	1
rs8177685	*IL2RA*	10	2	2
rs2476601	*PTPN22*	1	3	4
rs2274037	*IL2RA*	10	4	111
rs1569723	*CD40*	20	5	5
rs7559874	linkage	2	6	6
rs2416810	*TRAF1-C5*	9	7	65
rs7795093	association	7	8	3
rs1179766	*TRAF1-C5*	9	9	121

## Discussion

In this study, SE, the strongest genetic component to RA, was identified by both MDR and RF methods with similar prediction accuracy (MDR: 76%, RF: 72%). SE did not distinguish controls as well as cases using the RF method, which reflects the fact that nearly all of the cases are positive for SE but about half of the controls are also positive. SE alone is not sufficient to distinguish RA from "normal," but as additional genes were added, the prediction error was increasingly similar between cases and controls. We used the omnibus method to detect genes other than SE. All three methods detected *PTPN22 *as being important to RA susceptibility. *TRAF1-C5 *was identified by both the RF and omnibus methods and was suggested by the MDR two-locus analysis. These findings are consistent with the results from the single-SNP association analysis [4]. Thus, our study using real data demonstrates the ability of these high-dimensional screening methods to detect gene effects. The three methods have the advantage of allowing for both gene main effects and interactions to be tested because they consider multilocus genotypes. However, they cannot explicitly test significance of the interaction terms because there is no formal nested model testing. We used standard logistic regression to test the interaction terms but we did not find evidence of gene-gene interaction. It is likely that the strong main effect of SE limits our ability to detect both additional genes and the presence of gene-gene interaction effects.

Based on our analysis, we conclude that both MDR and RF are useful exploratory approaches for finding gene effects when many genes (or SNPs) are tested. The omnibus method is especially designed to test a gene (taking all of the SNPs in one gene as a whole group) in the presence of another known gene or risk factor. Thus, it was ideal in this setting because the SE is already known to have a strong effect in RA susceptibility. Genes identified by these exploratory methods can then be taken forward in replication studies and examined using more formal statistical modeling.

## Conclusion

For exploratory analysis of high-dimensional genotype data in complex diseases, MDR, RF, and a new omnibus method can all be used as tools to screen for genes of importance, whether they have main effects or are involved in interactions. These methods were all successful in identifying genes previously suggested for RA. However, they did not identify additional genes. The challenge of determining the precise genetic models of susceptibility will require other methods along with large numbers of study subjects.

## List of abbreviations used

GAW16: Genetic Analysis Workshop 16; MDA: Mean decrease in accuracy; MDG: Mean decrease in Gini; MDR: Multifactor dimensionality reduction; RA: Rheumatoid arthritis; RF: Random forests; SE: Shared epitope; SNP: Single-nucleotide polymorphism

## Competing interests

The authors declare that they have no competing interests.

## Authors' contributions

XL participated in study design, data management, performed the statistical analyses, and drafted the manuscript. YG, TKL QL, CF, and XRY participated in study design, statistical analyses, interpretation of results, and aided in drafting the manuscript. AMG and LRG provided direction concerning analysis and recommendations at each phase of the analysis. All authors have read and approved the final manuscript.
